# Emerging diseases--what now?

**DOI:** 10.3201/eid0403.980343

**Published:** 1998

**Authors:** G A Alleyne

**Affiliations:** Pan American Health Organization, Washington, D.C., USA.


### Emerging Diseases—What Now?

**George A. O. Alleyne**
Pan American Health Organization, Washington, D.C., USA

The Pan American Health Organization (PAHO) was born in 1902 out of concern for the spread of infectious diseases. The outbreak of cholera in Hamburg in 1892 and the epidemics of yellow fever in the Americas led to the decision to establish the International Sanitary Bureau with permanent headquarters in Washington. At the conference that made this historic decision in 1902, participating countries agreed to cooperate with each other and transmit to the bureau "all data of every character relative to the sanitary conditions of their ports and territories and furnish said Bureau every opportunity and aid for a thorough and careful study and investigation of any outbreaks of pestilential disease." All this was to be done to provide the "widest possible protection of the public health of each of the said republics and that commerce between said republics may be facilitated."

To a very large extent, we are still following the bureau's recommendations, only the list of pestilential diseases is shorter by one. Smallpox is no longer with us—and cholera, yellow fever, and bubonic plague are now among the emerging diseases. Cholera is far from disappearing. There were approximately 400,000 cases in the Americas in 1991. This number fell to 18,000 in 1997, but recent reports indicate that as a result of flooding caused by El Niño, the number of cases in Peru has increased dramatically this year. For the first 4 weeks of this year, 2,863 cases were reported compared with 174 for the same period last year and 3,500 for the whole of 1997.

Over the past 5 years, emerging diseases have caused intense concern and activity. The growth in international travel is a major factor. Statistics from the World Tourism Organization show that some 1 million persons per day traveled from their homes by air in 1995. International travel has increased every one of the past 10 years with an average increase of 5.5% per annum. Approximately 1.6 million people cross or recross the U.S.-Mexico border every day by land. Cholera did not spread between the Peruvian towns of Chancay and Chimbote by air travel, but by normal intercity traffic.

The spread of antibiotic resistance is another reason for the emergence of disease; the indiscriminate use of antibiotics is to blame. In the South, antibiotic abuse is facilitated by ready availability without a prescription. In some countries, local pharmacies stock and dispense antibiotics with the same facility as they do cough syrups. In one study of private pharmacies, 42% of the antibiotics were dispensed without prescription (1); in another study, only 23% were given with a physician's prescription (2).

The essential elements of a control strategy for addressing emerging infections are a surveillance system, strengthening the public health infrastructure (including enhancing laboratory capability), stimulation of research, and training of personnel. This strategy is difficult. However, a review of past surveillance activities provides specific lessons.

At the regional level, three disease surveillance systems (for foot-and-mouth disease, poliomyelitis, and measles) have worked and are working. An essential common feature is that surveillance leads to definitive action. For example, detection of cases of poliomyelitis (before the disease was finally eliminated from the Americas) automatically triggered a response. The report of a suspected case now causes resources to be mobilized to establish the validity of the report.

In addition, strong motivation undergirds surveillance. In the case of animal vesicular disease, there is the intense commercial interest behind the maintenance of the system and the possibility of eradication of foot-and-mouth disease. The commercial interest arises because elimination of the disease from the countries of the South represents a possibility of exporting beef worth billions of dollars. Interest rests not only with the national authorities; small communities actually drive the system. An estimated 70% of the cattle are owned by peasants, who each own 10 or fewer. Systematic regular feedback is necessary to maintain interest.

The surveillance systems for these diseases are based on the use of geographic coordinates to divide the countries into grids that represent the special unit in which the data are collected. Reports are sent by the local veterinary service to the Pan American Foot-and-Mouth Disease Center in Brazil. In recent years, a system has been developed for childhood illnesses that is as sensitive as that which reports animal diseases. The driving force behind the successful development and maintenance of the surveillance system for these childhood illnesses is the possibility of a finite end—eradication and the emotional pride that national health workers and politicians have in reaching this end.

Perhaps the most important aspect of successful surveillance systems is the presence of a credible coordinating international body. No effective international surveillance system can be mounted by a single country, no matter how well it is endowed. External energy, commitment, expertise, and persistence are necessary for such systems to function.

The technology of communication should not become the focus of our efforts. The surveillance and containment systems for smallpox depended on telegrams, telexes, and, I suspect, talking drums. "In India, the largest of the endemic countries, there were no fewer than 8,167 units reporting weekly to 397 district offices, which in turn reported to 31 state program offices and those to the national program office in New Delhi" (3). All this and more was sent to Geneva to be analyzed and reported back faithfully, without the benefit of electronic mail. New information technology is not an indispensable part of the solution.

It is challenging to our sense of superiority as a species to realize that diseases will always emerge. Changes in our social and physical ecology will almost certainly ensure the emergence of new or old diseases, and we are now more vulnerable to these diseases than before. Thus, strategies and policies must be able to be adapted to confront the inevitable new threats; the international community must avoid the peaks and valleys of action that accompany public interest in the exotic.

We have already begun to implement agreed-upon strategies in one particular area. To establish a system for surveillance of antibiotic resistance to enteric pathogens, we identified participating laboratories in 14 countries of the Americas. The next step was to standardize isolation techniques and review methods for measuring antibiotic sensitivity. We are applying an approach similar to the one that proved successful with the Pan American Regional Poliomyelitis Laboratory Network and have adopted "open regionalism"—establishing limited networks that may expand eventually and cooperate among themselves.

PAHO is also creating a functional network of laboratories in the greater Amazon Region to provide data on emerging infections. The participating laboratories' common objective will be the provision of accurate results, prompt sharing of information and research protocols, and a mechanism for rapid transfer of technology. However, the laboratories will need external support to sustain the system.

A strong global system for the application of strategies to control emerging diseases will not occur if the agreement on global action exists only in the sphere of surveillance. There is a fundamental need for other health professionals, in addition to microbiologists, to be convinced of the need for a global approach to some health issues.

The fear of infectious disease has been a powerful stimulus for global action. The successful global system for influenza is due partly to the coordinating efforts of the World Health Organization (WHO) and the work of the key collaborating laboratory centers. Involvement in these efforts keeps laboratories abreast of the latest developments in their special fields.

The need for global health coordination has been very much in the news; the appropriate body to perform that function is WHO. Most nations agree that they must assume responsibility for what are called essential public goods, e.g., immunization, provision of clean water. But some goods are public beyond national considerations, and no single nation can coordinate the availability of these international public goods.

International leadership goes beyond emerging diseases; indeed the success of a global effort to address the threat of these diseases depends largely on the wider perception of responsibilities for global coordination in health. Some believe that the global effort must focus on problems more common in the developing world and that global coordination is a mechanism for channeling resources from the rich to the less fortunate. However, all countries need to appreciate the benefits of global coordination of efforts such as those needed to address emerging diseases. Multilateralism is not antithetical to national interests or bilateral approaches. Success of this multilateral approach will require budgetary support. The annual regular budget of WHO is approximately US$420 million—14% of PAHO's budget. As Joshua Lederberg said, "Our thinking has been impoverished in terms of budget allocation for dealing with health on an international basis."

Some very successful efforts at global coordination in health have been disease or theme specific, and the "Special Program" approach has given some very good results. However, we should go beyond that and have a global health forum or council in which those agencies and institutions active or becoming increasingly active in health join with WHO in determining how to coordinate the various efforts. I would include in this forum representation from the multilateral financial institutions, the private sector, and nongovernmental organizations. Different spheres of interest and action would complement each other, which should help correct the current ad hoc theme-driven approach that continues to draw criticism.

PAHO has emphasized the benefit of a collective approach, and Panamericanism is one of the major underlying principles of the organization's work. For example, "Health Technology Linking the Americas," a concept that promotes the availability of simple effective technologies throughout the Americas, is a current initiative. Vaccines are one of the technologies emphasized.

In conclusion, we must promote the individual study of the nature and local means of control of emerging diseases. However, we also need a more collective approach at the regional, or even better, the global level—this approach is bound up with the support for global action on other fronts in health. The most powerful instrument we have is multipronged advocacy—advocacy is needed at the political and popular levels for this approach. The public must be engaged on a more regular basis to consider the truism that public health must be a concern of the public. This advocacy has to use some specific examples of those matters that affect the public's health so that emerging diseases are not seen as a threat only on television.

### References

1. Brieva J, Danhier A, Villegas G, Yates T, Pérez H. Modalidades del uso de antibióticos en Concepción, Chile. Boletín Oficina Sanitaria Panamericana 1987;103(4):363-72.
2. López R, Kroeger A. Morbilidad y medicamentos en Perú y Bolivia. Universidad Peruana, Cayetano Heredia, Lima, Perú. Acción para la salud, Chimbote, Perú. Ministerio de Salud, La Paz, Bolivia. Universidad de Heidelberg, Alemania, 1990.
3. Fenner F, Henderson DA, Arita I, Jezek Z, Ladnyi ID. Smallpox and its eradication. Geneva: World Health Organization; 1988. p. 497.
